# Opioid exposure on the preterm brain: qualitative and quantitative MRI analysis

**DOI:** 10.1007/s44253-025-00105-1

**Published:** 2025-12-29

**Authors:** S. de Munck, F. Savvopoulos, J. Dudink, M. H. G. Dremmen, L. C. C. Toussaint-Duyster, N. Bouw, M. J. Vermeulen, N. E. M. van Haren, G. E. van den Bosch

**Affiliations:** 1 Department of Paediatric Surgery, Erasmus MC Sophia Children’s Hospital, Rotterdam, The Netherlands; 2 Department of Child and Adolescent Psychiatry/Psychology, Erasmus MC Sophia Children’s Hospital, Rotterdam, The Netherlands; 3 Department of Neonatology, Wilhelmina Children’s Hospital, University Medical Centre Utrecht, Utrecht, The Netherlands; 4 Department of Radiology and Nuclear Medicine, Erasmus MC, Rotterdam, The Netherlands; 5 Department of Orthopaedics, Section Physical Therapy, Erasmus MC, Rotterdam, The Netherlands; 6 Department of Neonatal and Paediatric Intensive Care, Division of Neonatology, Erasmus MC Sophia Children’s Hospital, Rotterdam, The Netherlands

## Abstract

**Aim:**

To explore whether opioid exposure (morphine or fentanyl) in the neonatal intensive care unit is associated with altered neurodevelopmental outcome in preterm infants, and whether this association is modified by qualitative (brain lesions) or quantitative (volumetric) brain MRI measures obtained around 30 weeks postmenstrual age (PMA).

**Method:**

This retrospective cohort study included 280 infants born ≤ 30 weeks of gestation (2008–2016), scanned at 1.5T before 33 weeks PMA. Brain injury was scored by paediatric (neuro)radiologists; brain volumes were obtained via automated segmentation (dHCP-pipeline). Neurodevelopmental outcome was assessed at age 2 (Bayley-III) and age 5 (WPPSI-III, M-ABC-2). Associations between opioid exposure, brain measures, and outcome were examined using multivariable regression and interaction models.

**Results:**

Opioid exposure was associated with lower cognitive scores at age 2, but not with IQ at age 5. Motor scores at age 5 were lower in children exposed to opioids. Opioid administration was associated with increased risk of intraventricular haemorrhage and post haemorrhagic ventricular dilatation, but not with brain volumes. No significant interaction between opioid administration and brain injury or volume on outcome was demonstrated.

**Interpretation:**

Opioid exposure in preterm neonates is associated with poorer neurodevelopmental outcome in early childhood, but not with early brain injury or structural volumetrics on MRI after birth. Long-term follow up and longitudinal brain imaging may provide greater insight in the association between opioid administration and poor neurodevelopmental outcome. Thus far, quantitative or qualitative brain metrics around 30 weeks PMA have not been proven useful.

**Supplementary Information:**

The online version contains supplementary material available at 10.1007/s44253-025-00105-1.

## Introduction

During the end of the second and the third trimester of pregnancy, the brain of a foetus is rapidly developing [1]. For extremely and very preterm born children (< 32 weeks gestational age), this important phase of brain development does not take place in the safe environment of the uterus, but in the hectic atmosphere of a neonatal intensive care unit (NICU). Preterm birth is associated with increased vulnerability to disrupted brain development, including acquired brain injuries such as haemorrhage and infarction, which may contribute to later neurodevelopmental impairments depending on their severity and location [2–5]. However, the absence of visible lesions does not imply typical development. Subtle alterations in neurodevelopmental trajectories, such as impaired brain growth or aberrant maturation of structural and functional connectivity, may ultimately manifest as cognitive, motor, behavioural, or socio-emotional difficulties [6–15], highlighting the need for early risk stratification and targeted intervention.

Several potential risk factors for atypical brain development in preterm born children have been identified, including postnatal exposure to stress and pain, which are known to negatively influence brain development and outcome [16, 17]. However, administration of opioids to relieve stress and pain might also affect neurodevelopment [18–22]. Nevertheless, long term follow up studies on neonatal opioid exposure have showed contradictory results, which may be explained by differences in for example gestational age, pre-emptive or therapeutic use, cumulative exposure or age during follow up [9, 18, 23–26]. Yet, evidence suggests that opioid receptors in the brain play a direct role in neuronal development, although the exact pathogenesis and pathophysiology have not been fully elucidated [27]. Animal studies showed that opioids alter the dendritic architecture, reduce neuronal and µ opioid receptor density, and affect neurodevelopmental outcome [27, 28]. Exposure to opioids in preterm infants has been associated with reduced cerebral growth on MRI at term-equivalent age, a finding that may underlie later neurodevelopmental difficulties [18, 29]. In addition, opioid exposure has been linked to the presence of brain lesions, such as intraventricular haemorrhage and white matter injury [30, 31]. However, this relationship is likely bidirectional: infants with brain injury often require more intensive care and are therefore more likely to receive opioids. As such, opioid administration may reflect illness severity rather than function as a primary causal factor for brain injury.

It is nonetheless valuable to understand whether preterm born children who are exposed to opioids are more susceptible to poor neurodevelopmental outcome when compared to non-exposed preterm born children, and whether aberrant qualitative and quantitative characteristics of the brain influence this association. We will therefore explore the effects of opioids on neurodevelopmental outcomes (i.e. motor and cognitive performance) at two and five years corrected age, and on qualitative and quantitative measures of the brain. Next, we explore whether the relation between brain measures (lesions, volumes) and outcome is different for infants exposed to opioids compared to those not exposed to opioids.

In this explorative study, we hypothesize that (extremely to very) preterm born children exposed to opioids in the first weeks of life (i.e. morphine or fentanyl) are at risk for delayed neurodevelopmental outcomes at two and five years of age and a higher burden of qualitative and quantitative brain deviations. We also explore if the association between brain measures around 30 weeks post menstrual age (PMA) and neurodevelopmental outcome differs between neonates who were or were not administered opioids.

## Methods

### Patients

We included all preterm infants born at ≤ 30 weeks of gestation, admitted to the level III-IV NICU of Erasmus MC Sophia Children’s Hospital between January 2008 and October 2016, who underwent routine brain MRI before 33 weeks postmenstrual age (PMA) [32]. The 33-week PMA cutoff was chosen to minimize developmental variability across the cohort. Neonates were not eligible for this study in case of major congenital anomalies or syndromes known to affect neurodevelopmental outcome.

The study protocol was reviewed by the Institutional Review Board of Erasmus Medical Centre, which determined that, due to the observational nature of the study, formal ethical approval under the Dutch Medical Research Involving Human Subjects Act (MEC-2018-1142) and written informed consent were not required. Upon clinical admission, parents were informed that anonymized data from medical records would be used for research and registry purposes. If parents chose to opt out, their data and images were excluded from the study.

### Clinical characteristics

Perinatal characteristics, retrieved from medical records, included twin or triplet pregnancy (yes/no), mode of delivery (caesarean section or vaginal), gestational age at birth (weeks), birthweight (grams and z-score; Fenton [33]), sex, Apgar score at five minutes, inborn (our hospital, yes/no), length of NICU stay (days), need for invasive mechanical ventilation (yes/no), duration of invasive mechanical ventilation (days), sepsis during NICU stay (yes/no, defined as positive blood culture), surgical intervention needed (yes/no, any abdominal surgery and clipping of ductus arteriosus). Socioeconomic status was based on maternal education level (MEL, low-to-middle or high, based on the International Standard Classification of Education (ISCED) [34]).

The data on administration of the most frequently prescribed opioids at our NICU, morphine and fentanyl, were collected for two time windows: throughout the NICU admission until the moment of scanning (yes/no), and throughout the NICU admission until term equivalent age (TEA, yes/no). Individual cumulative dosage until MRI scan, as well as the average administered dosage per kilogram per day were calculated. To complete the overview of analgesic and sedative medication, we recorded if children had received paracetamol, midazolam, and/or methadone (yes/no).

### Neurodevelopmental outcome

The included children attended the standardized national neonatal follow up program at our out-patient clinic. Neurodevelopmental assessment was performed at 2 years corrected age using the Dutch version of the Bayley Scales of Infant and Toddler Development, 3rd edition (Bayley-III, cognitive and motor scale) [35], and at age 5 using the Dutch version of the Wechsler Preschool and Primary Scale of Intelligence, 3rd edition (WPPSI-III-NL, (full scale IQ (FSIQ), verbal IQ (VIQ), performance IQ (PIQ) and performance speed (PS)) [36] and the Movement Assessment Battery for Children, 2nd edition (M-ABC 2, total score, balance skills, ball skills and manual dexterity)) [37]. Scores below − 1 SD (Bayley-III and WPPSI: <85; M-ABC: <7) were considered below the normative range. All assessments were conducted by trained and certified paediatric psychologists or physiotherapists.

### Neuroimaging

All MRI scans were performed with a 1.5T GE EchoSpeed scanner (General Electric Healthcare Technologies, Waukesha, Wisconsin, USA). For an overview of our MRI procedures, we refer to our previously published articles and Supplemental information 1 and 2 [32, 38].

### Qualitative analysis

Brain injury was scored independently by paediatric (neuro)radiologists and neonatologists using T1 or T2 weighted sequences. Lesions included intraventricular haemorrhage (IVH) grade I-III (classification by Volpe et al. [39]), post haemorrhagic ventricle dilatation (PHVD) for which intervention was needed, venous infarction, punctate white matter lesions, white matter cysts (unilateral), periventricular leukomalacia (PVL, bilateral: both cystic and non-cystic) and cerebellar haemorrhage [40]. These lesions were included as they are considered to possibly affect neurodevelopmental outcome [41]. Note that scoring of lesions took place right after the scan was made, and might therefore not reflect the final diagnosis, as lesions could still have progressed over time (e.g. from IVH grade 1 to IVH grade 3 or development of PHVD after the scan).

### Quantitative analysis

Axial fast-spin echo (FSE) T2-weighted scans were used for volumetric segmentation. Scans with major artefacts or lesions precluding accurate segmentation were excluded. Brain scans were segmented into eight volumes using the dHCP structural pipeline: cortical grey matter (CGM), deep grey matter (DGM), white matter (WM), hippocampi and amygdala (together, HA), cerebellum (CBL), brainstem, ventricles, and cerebrospinal fluid (CSF). Intracranial volume was defined as the sum of all the eight volumes. Total brain volume (TBV) was defined as intracranial volume minus ventricles and CSF outside the ventricles. A quality check of the segmentations was done by visual inspection (SM, FS and GB). Scans with incorrect segmentations were excluded. When only specific regions were affected, multiple imputation was used to estimate missing volumes. For details regarding processing and volume calculations, refer to Supplemental information 3.

### Statistical analysis

Mann-Whitney U tests and Student t-tests were used to compare continuous data of patients with and without opioids; Chi-square tests and Fisher’s exact tests were used for categorical data. One sample t-tests were used to compare continuous test results with normative scores. Multivariable regression analyses were performed to assess associations when assumptions for linear regression were met. Given the explorative nature of this study, a p-value of < 0.05 (two-sided) was considered statistically significant. Statistical analyses were performed using SPSS, Version 28 (IBM SPSS Statistics, IBM Corporation, Armonk, NY).

### Opioid exposure and neurodevelopmental outcome

Neurodevelopmental outcome scores across all domains were compared to normative data to evaluate the overall cognitive and motor development of the cohort. Likewise, scores were compared between patients who received opioids and those who did not.

### Opioid exposure and brain measures

A chi-square test was used to examine whether opioid exposure before MRI was associated with the frequency of brain lesions, by comparing the number of patients with specific lesion types (e.g., IVH, PVL) between the exposed and unexposed group. To study the association between opioid use (yes/no) and brain volumes (TBV, CGM, DGM, WM, HA, CBL), we performed regression analysis correcting for intracranial volume, GA at birth, PMA at scan and sex. The selection of covariates was based on clinical relevance and previously demonstrated associations [42].

### Opioid exposure and interaction between brain measures and neurodevelopmental outcome

For brain lesions that showed a significant difference in frequency between infants who received opioids and those who did not, we performed multivariable regression analyses to study the association between outcome scores and brain lesions, adding GA at birth, PMA at scan, duration of invasive mechanical ventilation, MEL, opioids pre-TEA (yes/no) as covariates. Analyses were repeated while adding an interaction term of administration of opioids before TEA (yes/no) and brain lesions to investigate if the association between outcome and brain lesions differed between infants who were or were not administered opioids.

If an association between opioids (yes/no) and outcome scores was found, explorative regression analysis was performed with an interaction term of administration of opioids before TEA (yes/no) and TBV, and with an interaction term of administration of opioids before TEA (yes/no) and HA. The latter was included as opioid receptors are strongly expressed in the limbic system [43]. Analyses were adjusted for GA at birth, duration of invasive mechanical ventilation, PMA at scan and MEL.

## Results

### Patients

**Fig. 1:**
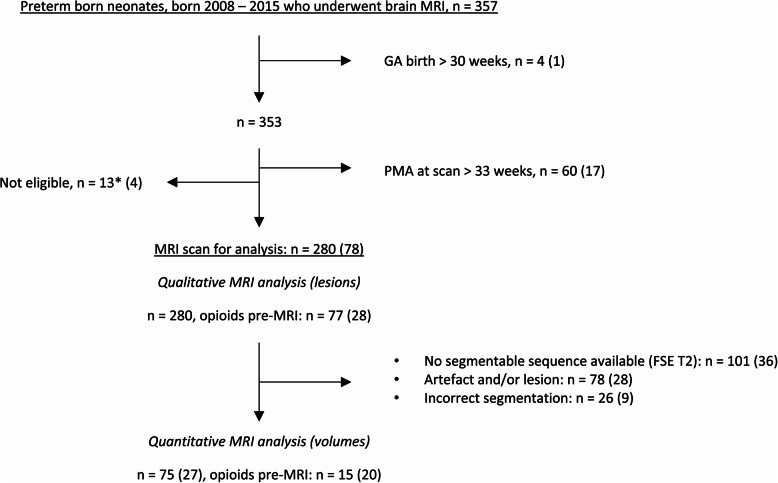
Inclusion flowchart

Three hundred-fifty-seven infants met the inclusion criteria. MRI scans of 280 neonates were included in the final analysis, eligible for qualitative analysis, quantitative analysis, or both. Segmented brain volumes were obtained from the scans of 75 infants. For an overview, see the flowchart in Fig. 1. As infants with clearly visible brain lesions were excluded from volumetric analyses, volumetric analyses were performed on a subset of infants with relatively normal brain structure, and results should be interpreted in this context.

**Fig. 2:**
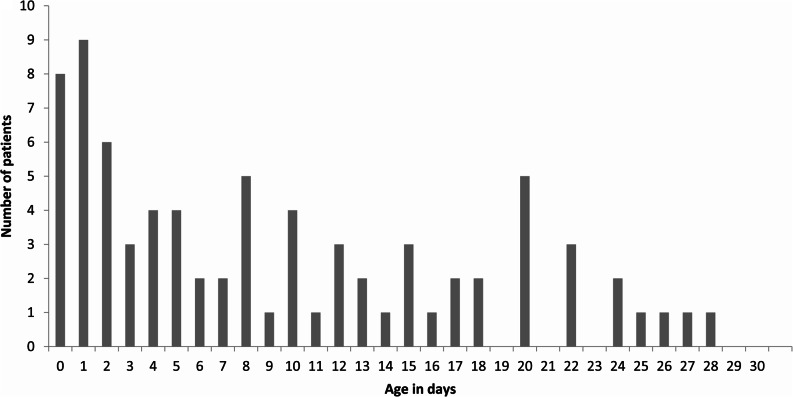
Age at first opioid administration Age at first opioid administration (morphine or fentanyl), in all neonates who received opioids before MRI (*n*=77). The x-axis indicates postnatal day of first opioid dose, the y-axis represents the number of patients

Opioids were administered prior to MRI to 77 neonates (28%) and to 93 neonates before TEA (33%). For the 280 included infants, median GA at birth was 27 weeks and median duration of NICU stay was 35 days. See Table 1 for an overview of the background and clinical characteristics. In the neonates who received opioids prior to MRI, the opioids were administered on median postnatal day eight. (Fig. 2). For data on opioid administration, see Table 2.

**Table 1:** Clinical characteristics

	**Opioids pre-MRI** (*n* = 77)		**No opioids pre-MRI** (*n* = 203)				
	**n or median**	**(%) or [IQR]**	**n or median**	**(%) or [IQR]**			**Difference** p*
Sex							
*Male*	48	(62)	97	(48)			0.03
*Female*	29	(38)	106	(52)			
Mode of delivery							
*Vaginal birth*	41	(53)	90	(44)			
*Caesarean section*	36	(47)	113	(56)			0.22
Twin or triplet pregnancy	22	(29)	54	(27)			0.76
Inborn	65	(84)	184	(91)			0.14
APGAR score at 5 min	7	[6–8]	8	[7–9]			0.02
Gestational age at birth, *weeks*	26.1	[24.9–27.1]	27.7	[26.4–28.3]			< 0.001
Birthweight, *grams*	840	[703–995]	990	[863–1158]			< 0.001
Z-score Fenton 2013 Preterm Growth Chart	0.3	[−0.4–0.8]	0.3	[−0.4–0.9]			0.95
Length of NICU stay, *days*	59	[44–75]	29	[19–40]			< 0.001
Need for invasive mechanical ventilation	76	(100)	134	(67)			< 0.001
Duration of invasive mechanical ventilation, *days*	21	[11–27]	2	[0–7]			< 0.001
Sepsis	32	(42)	44	(22)			< 0.001
Surgical intervention needed							
*Abdominal surgery*	19	(25)	9	(4)			< 0.001
*Clipping of patent ductus arteriosus*	30	(39)	7	(3)			< 0.001
Maternal education level							
*Low-middle*	38	(49)	100	(49)			
*High*	23	(30)	66	(33)			0.78
Postmenstrual age at MRI, *weeks*	30.6	[30–32]	30.1	[29.9–30.7]			< 0.001

*IQR* Interquartile range, *NICU* Neonatal intensive care unit, *MRI* Magnetic resonance imaging

**Table 2:** Opioid administration

	**n or median**	**(%) or [IQR]**	**min - max**
Opioids only pre-MRI	40	(14.3)	
Opioids pre-MRI and post MRI – pre-TEA	37	(13.2)	
Opioids only post MRI – pre-TEA	16	(5.7)	
Morphine until MRI	72	(25.7)	
*cumulative dosage mcg/kg*	938	[517–1566]	63–8576
*days administered*	5	[4–9]	1–30
*mcg/kg/day*^a^	172	[133–220]	64–351
Fentanyl until MRI	59	(21.1)	
*cumulative dosage mcg/kg*	7.1	[4.1–17]	0.5–333.8
*days administered*	1	[1–2]	1–30
*mcg/kg/day*	5.7	[3.6–8.8]	0.5–263
Paracetamol until MRI	29	(10.4)	
Midazolam until MRI	16	(5.7)	
Methadone until MRI	0	(0)	
Morphine until TEA	87	(31.1)	
*cumulative dosage mcg/kg*	986	[508–2054]	63–9913
*days administered*	6	[4–12]	1–48
*mcg/kg/day*	168	[141–220]	55–363
Fentanyl until TEA	74	(26.4)	
*cumulative dosage mcg/kg*	9.5	[4.6–24.2]	0.5–1717
*days administered*	1	[1–2.25]	1–34
*mcg/kg/day*	5.8	[3.7–9]	0.5–263
Paracetamol until TEA	63	(22.5)	
Midazolam until TEA	16	(5.7)	
Methadone until TEA	1	(0.4)	

*MRI* Magnetic resonance imaging, *TEA* Term equivalent age

Opioids: morphine and/or fentanyl

^a^calculated over number of days during which opioid was administered

### Opioid exposure and neurodevelopmental outcome

At age two, Bayley-III cognitive scale scores were unavailable for 45 children (16%) due to loss to follow-up, while the motor scale was not assessed in 126 children (45%). At age five, the WPPSI-III could not be conducted in 135 (48%) of the eligible children, and 214 children (76%) were not assessed with the M-ABC 2. Main reasons for the loss to follow up were follow up elsewhere, refusal, migration, and no-show without a known reason.

Cognitive scores of the Bayley-III (*n* = 215) were significantly lower in children who were exposed to opioids before TEA compared to those who had not been exposed (*p* = 0.008). No significant differences were found in motor scores of the Bayley-III between these two groups.

At age five, FSIQ, PIQ, VIQ and PS (WPPSI-III, *n* = 146) did not differ significantly between children exposed and those not exposed to opioids. Scores for full scale IQ and all three subdomains fell significantly below the norm in those who had not been exposed to opioids, whereas this was only the case for full scale IQ and processing speed in the opioid group.

As for motor skills (M-ABC 2, *n* = 66), children who had been exposed to opioids scored significantly lower on ball and balance skills compared to those who had not (*p* ≤ 0.03). Details on outcome scores for total cohort and opioid group (yes/no) are shown in Supplemental information 4.

### Opioid exposure and brain measures

In patients exposed to opioids before the MRI, significantly more cases of IVH (any grade) and PHVD were reported compared to patients who were not exposed to opioids (*p* = 0.001). The frequencies of venous infarction, PWML, white matter cysts, PVL and cerebellar haemorrhage did not differ significantly between groups (Table 3).

**Table 3:** Brain injury

	Opioids pre-MRI, (*n* = 77)		No opioids pre-MRI, (*n* = 203)			
	n	(%)	n	(%)	p	
Intraventricular haemorrhage, any grade	41	(53.2)	66	(32.5)	**0.001**	
Intraventricular haemorrhage, grade						
*1*	22	(53.7)	42	(63.6)		
*2*	11	(26.8)	17	(25.8)		
*3*	8	(19.5)	7	(10.6)	0.39	
Venous infarction	9	(11.7)	11	(5.3)	0.11	
Post haemorrhagic ventricle dilatation^a^	5	(6.5)	3	(1.5)	**0.04**	
Punctate white matter lesions	5	(6.5)	22	(10.8)	0.37	
White matter cysts, unilateral	2	(2.6)	5	(2.5)	1.00	
Periventricular leukomalacia	0	(0)	6	(3.0)	0.19	
Cerebellar haemorrhage	11	(14.3)	23	(11.3)	0.54	

^a^For which intervention was needed

Bold: significant difference between groups

There were no significant differences in any of the brain volumes between patients exposed versus not exposed to opioids before MRI (Table4.).

**Table 4:** Multiple linear regression analysisbetween brain volumes and opioid administration (yes/no)

		Opioids pre-MRI^a^
Total brain volume	Unstandardized B	1.14
	95% CI	−2.14–4.42
	p	0.50
Cortical grey matter	Unstandardized B	0.90
	95% CI	−0.67–2.46
	p	0.26
White matter	Unstandardized B	0.76
	95% CI	−2.19–3.70
	p	0.61
Deep grey matter	Unstandardized B	−0.27
	95% CI	−0.78–0.23
	p	0.29
Hippocampus, amygdala	Unstandardized B	0.03
	95% CI	−0.04–0.10
	p	0.43
Cerebellum	Unstandardized B	−0.14
	95% CI	−0.62–0.34
	p	0.57

Volumes in millilitres

*CI* Confidence interval, *MRI* Magnetic resonance imaging

^a^Adjusted for intracranial volume, GA at birth, PMA at MRI and sex

### Opioid exposure and interaction between brain measures and neurodevelopment

We found no significant association between IVH (any grade) and neurodevelopmental outcome scores in infants who were not exposed to opioids (all p-values ≥ 0.351). None of the interactions reached significance (all p-values ≥ 0.06), indicating that the association between IVH and these measures of neurodevelopmental outcome in infants exposed to opioids did not differ from infants not exposed (Table 5).

**Table 5:** Multiple linear regression analysis between opioid administration (yes/no), intraventricular haemorrhage and neurodevelopmental outcome scores

		**IVH**^**a**^	IVH*opioids^**b**^
Bayley-III cognitive score (*n* = 215)	Unstandardized B	0.89	−6.55
	95% CI	−3.01–4.79	−13.38 – −0.29
	p	0.66	0.06
Bayley-III motor score (*n* = 154)	Unstandardized B	−2.02	−5.22
	95% CI	−6.28–2.25	−12.41–1.96
	p	0.35	0.15
WPPSI TIQ (*n* = 133)	Unstandardized B	−4.22	3.87
	95% CI	−10.93–1.96	−6.54–14.27
	p	0.18	0.46
M-ABC 2 total score (*n* = 66)	Unstandardized B	−0.37	−0.70
	95% CI	−2.51–1.77	−4.32–2.93
	p	0.73	0.70

All adjusted for gestational age at birth, duration of invasive mechanical ventilation, maternal education level, post menstrual age at scan

*N* Number of cases used for analysis, *CI* Confidence interval, *WPPSI* Wechsler Preschool and Primary Scale of Intelligence, *TIQ* Total IQ, *M-ABC 2* Movement Assessment Battery for Children second edition, *TEA* Term equivalent age, *IVH* Intraventricular haemorrhage

^a^B represents the change in neurodevelopmental outcome score in infants with IVH who were not exposed to opioids

^b^B represents the change in neurodevelopmental outcome score infants with IVH exposed to opioids compared to those not exposed to opioids with IVH

We found no significant association between TBV (*p* = 0.525) nor HA (*p* = 0.506) and neurodevelopmental outcome scores in infants who were not exposed to opioids. In infants who were exposed to opioids, the interaction terms of TBV and HA with opioid administration were not significantly associated with Bayley-III cognitive score (respectively*p* = 0.0.193 and *p* = 0.679), indicating that the association between both TBV and HA with cognitive outcome is not negatively different in neonates who were exposed to opioids (Table 6.).

**Table 6:** Multiple linear regression analysis between opioid administration (yes/no), brain volumes and Bayley-III cognitive score

		TBV	TBV*opioids	HA	HA*opioids
Bayley-III cognitive score	Unstandardized B	−0.07^a^	0.33^b^	8.72^a^	10.76^b^
	95% CI	−0.28–0.14	−0.17–0.83	−16.95–34.38	−40.19–61.72
	p	0.53	0.19	0.506	0.68

All adjusted for gestational age at birth, duration of invasive mechanical ventilation, maternal education level, post menstrual age at scan.

*CI* Confidence interval, *TBV* Total brain volume, *HA* Volume of hippocampi and amygdala

Volumes in millilitres

^a^B represents the change in Bayley cognitive score (−0.011) for 1 ml increase in TBV volume in infants not exposed to opioids

^b^B represents the excessive change in outcome on Bayley n infants exposed to opioids compared to those not exposed to opioids with an increase of 1 ml of TBV/HA volume

## Discussion

We demonstrated that opioid administration in preterm born children was negatively associated with cognitive outcome scores at two years corrected age, and with lower on ball and balance skills at age five, but no significant association was observed with IQ scores at age five. Moreover, opioid administration was associated with a higher incidence of IVH and PHVD, but not with brain volumes at 30 weeks PMA. No significant interaction between opioid administration and brain injury or volume on outcome was demonstrated.

Previous studies have extensively studied the effect of opioid use on brain development and neurodevelopmental outcome in preterm born children [18, 21, 29, 44]. Yet, few studies have investigated the interrelationships between opioid administration, brain measures and neurodevelopmental outcome, and studied brain development as early as 30 weeks PMA. By using longitudinal MRI data, Zwicker et al. found that morphine exposure was associated with poorer neurodevelopmental outcome at 18 months, mediated by reduced cerebellar growth between 30 and 40 weeks PMA [18]. Unfortunately, we lack longitudinal MRI data and are therefore not able to calculate brain growth or decline over time, which hampers comparison between cohorts.

### Opioids and neurodevelopmental outcome

We found that opioid use was negatively associated with cognitive outcome at two years of corrected age, but not with IQ scores at five years of age. This aligns with previous research, which has reported similar results: opioid effects on cognition possibly tend to fade over time [9, 19, 24, 25, 44, 45]. However, the studies by De Graaf et al. in an older cohort from our department (children born between 2000 and 2002, while children in current cohort were born between 2008 and 2015), found a significant negative effect of morphine on the ‘visual analysis’ IQ subtest at five years of age, although not on the other tests [25]. Important differences between both cohorts may explain why the older cohort was more vulnerable to developmental delays at five years of age compared to the current cohort. In the earlier cohort morphine was administered pre-emptively, whereas in our cohort it was given only in response to pain. Thus, advances in neonatal care, specifically related to pain management over time, may explain that no neurodevelopmental deviations were found in the more recent cohort. We hypothesize that a better opioid-pain balance in our cohort may have led to better outcomes. Future large-scale studies are needed to examine in greater detail the delicate balance between adequate pain management and potential effects of analgesic exposure on neurodevelopmental outcomes.

The previously described cohort was also scanned at 10 years of age, which study reported associations between prematurity, opioid exposure, neonatal pain and brain volumes, but no major effects on neuropsychological functioning [9]. These findings, consistent with earlier work by de Graaf et al., suggest that neonatal morphine does not adversely affect later neurocognitive outcomes in preterm children without early brain injury. However, given the small sample size, larger MRI studies were warranted, providing the rationale for the present study.

Yet, a recent study by Luzzati et al. found that morphine administration was associated with lower IQ scores five years of age [21]. The lower mean gestational age of that cohort compared to our cohort might have caused the discrepancy in findings. However, this does not explain their finding that morphine administration was not associated with motor or cognitive outcomes of the Bayley-III at two years of age.

Together these findings suggest that both the type of neurodevelopmental outcome test as well as the age at which it is assessed should be considered while interpreting results. The contradicting results reflected in previous literature and our study, together with the lack of outcome data on specific cognitive domains across different ages, highlight the need for extensive long-term follow up to study the developmental effects of opioids, preferably until late adolescence or early adulthood.

In contrast to cognitive function, we found significantly lower motor function scores at age five, reflected in lower ball and balance skills among preterm neonates who had received opioids compared to those who had not. Similar findings were reported by Mills et al. [44], although their associations lost significance after adjusting for multiple factors. Given the cerebellum’s critical role in motor coordination and its previously demonstrated vulnerability to opioid exposure [18, 46], one might expect an association between cerebellar volume, opioid exposure, and lower motor scores. Yet, despite the significant relationship between opioid exposure and lower motor function at five years of age, we did not observe an opioid-related effect on cerebellar volume at 30 weeks postmenstrual age.

### Opioids and brain injury

Our finding that opioid use pre-MRI is associated with IVH (any grade) is in line with previous studies. Opioid use has been commonly related to IVH, white matter lesions and cerebellar injury [21, 31, 45, 47]. Opioids may cause decreased arterial blood pressure [48], and thus IVH in case of underdeveloped cerebral autoregulation [49]. Although IVH usually occurs within the first few days of life in preterm born neonates [41], 49% of neonates in our cohort were administered opioids later than the first ten days of life, arguing against opioids being a direct cause of IVH. IVH could instead lead to discomfort necessitating opioid administration, making IVH the cause rather than the effect. This interpretation is supported by a recent large retrospective study, which found no association between early-phase opioid use and IVH in extremely preterm infants [50]. IVH usually occurs in the most critically ill neonates, who often have complex underlying pathophysiology and therefore receive more opioids compared to less sick peers. However, due to the lack of a severity of illness score, it was difficult to adequately account for this factor in our analyses. Consistently, PHVD was also associated with opioid use. A direct causal relationship between opioid administration and PHVD appears unlikely, as opioid use in this context is often necessitated by the need for neurosurgical interventions related to PHVD itself (e.g., placement of a Rickham reservoir or ventriculoperitoneal drain), for which adequate analgesia is essential. Future studies incorporating longitudinal neuroimaging during the early postnatal period are needed to clarify the temporal and causal dynamics of this association.

### Opioids and brain volumes

Although we found no association between opioid administration and brain volume, this may be due to the timing of our scans at 30 weeks PMA. Other studies, which performed imaging at term-equivalent age (40 weeks PMA), have reported associations between opioid exposure and reduced brain and cerebellar volumes [29, 31], potentially explaining the discrepancy in findings.

This suggests that the effects of opioid administration on the brain cannot be visualised as early as at 30 weeks. Also, a consequence of scanning this early is the small window of opioid exposure prior to the scan. Indeed, in our cohort, 53 patients were still administered opioids after the MRI scan was made, while 40 patients only received opioids in the timeframe before the MRI. In those 40 patients, opioid administration could have happened only days before scanning, and no visible effects of opioids administered on the brain can yet be expected. Moreover, scanning at this early stage potentially introduces a selection bias, as some of the eligible neonates were still too sick and clinically unstable at 30 weeks GA to undergo a scan. It has previously been demonstrated that, to predict long term outcome, a cerebral MRI is preferably made at TEA [51], which potentially also applies for investigating the effect of opioids on the brain.

Again, a solution would be to conduct longitudinal brain scanning, at 30 weeks GA, TEA and beyond to establish the influence of opioids on brain development.

### Pain versus opioids

Pain and stress, commonly experienced by preterm neonates during NICU stay, are known to negatively affect brain development and neurodevelopmental outcome [16, 17, 26, 52–54]. In contrast, studies in rodents have shown that morphine might protect the brain from detrimental changes in development caused by pain [55, 56]. As data on experienced pain and stress were not systematically collected, we were unable to assess the balance between the potential neurodevelopmental impact of untreated pain and the possible iatrogenic effects of opioid administration [57]. For example, Selvanathan et al. demonstrated that morphine administration modified associations between pain and motor scores at 18 months of age, and more specifically that greater exposure to pain was associated with poorer outcome scores in children with no or long exposure, but not in those with short exposure to morphine, suggesting an optimal treatment approach [20].

### Strengths and limitations

Strengths of this study include the relatively large cohort size and the multifactorial design, incorporating both qualitative and quantitative MRI assessments alongside neurodevelopmental outcome data from standardized follow-up. However, imaging was performed in a routine clinical setting without a standardized research protocol, which limited the proportion of scans suitable for volumetric analysis. In addition, pain exposure could not be quantified, as validated pain assessment tools such as the COMFORTneo scale were not consistently implemented during the study period. We also lack a severity of illness measure, such as the CRIB score. Thus, the question remains whether opioids are the direct cause of brain injury and lower neurodevelopmental outcome scores, or rather a proxy for pain or severity of illness. We used the duration of invasive mechanical ventilation instead, as it most closely reflects the clinical condition of an infant in the ICU.

The absence of SWI sequences in our MRI protocol may have led to a slight underestimation of lesion count in our cohort. However, this likely pertains only to the smallest lesions that are not visible on T1- or T2-weighted images and is therefore considered clinically less relevant.

For the quantitative analyses concerning brain volumes, we could only include the scans of patients that did not have any significant brain lesions, as this would affect the registration to a template and the segmentation of the regions of interest. As a result, we included the neonates with relatively healthy brains for the quantitative analyses, as the sickest neonates more often suffer from brain injury [41].

During the study period, opioids were not administered pre-emptively, but at the discretion of the attending NICU professionals, in accordance with the pain protocol in our centre. Morphine was generally the first-line opioid in our unit, while fentanyl was primarily used as rescue medication or for procedural analgesia, such as during surgical interventions. Over time, evolving clinical insights have led to changes in local practice regarding both morphine dosing and the indications for fentanyl use.

Furthermore, we chose to only use a binary estimate of opioid administration (yes/no) in our analyses, instead of duration of administration or cumulative dosage. This is relevant, as Puia-Dumitrescu et al. found that infants with prolonged opioid exposure (>7 days) had lower Bayley-III scores at two years of age, while outcome scores of neonates who were only briefly exposed to opioids did not differ from outcome scores of neonates without opioid exposure [19].

For this explorative study, we chose to only study the effect of opioids on neurodevelopmental outcome and brain measures, as they are most used in our centre. Midazolam, of which previous studies have demonstrated a detrimental effect on neurodevelopment [19, 58], is also administered in our centre, but only in 5.7% of the patients in our cohort. We considered these numbers too small to run valid and reliable analyses on.

No power calculation was conducted in advance due to the exploratory design of this study, and no correction for multiple testing was applied. Therefore, effect estimates should be interpreted with caution.

### Future research

For future studies, we suggest emphasising the interplay between pain and opioid exposure. Nowadays, pain and its management are more closely monitored, enabling researchers to investigate this balance by using validated, standardized pain assessment scales, incorporating multimodal pain monitoring, and structural recording of administered opioid dosages. For MRI we recommend a standardized scanning protocol, with scans being made at least at TEA. These should be related to long-term outcome, particularly through regular assessments across multiple cognitive domains throughout childhood.

## Conclusion

This study examined the association between early opioid exposure and neurodevelopmental outcome in preterm infants and explored whether this relationship was modified by brain injury or brain volume assessed around 30 weeks postmenstrual age. While opioid exposure was associated with lower cognitive and motor outcomes in early childhood, no interaction was found for either brain injury or total and regional brain volumes at this early time point. It remains unclear whether the association is driven by opioid exposure itself or by illness severity, which is often correlated with higher opioid use and poorer cognitive and motor outcomes. Our findings suggest that conventional MRI at 30 weeks PMA may have limited sensitivity for detecting structural correlates of opioid-related neurodevelopmental risk. It is possible that microstructural alterations—such as impaired white matter maturation—occur in response to opioid exposure, which may only be detectable using advanced techniques such as diffusion tensor imaging (DTI). Longitudinal neuroimaging incorporating DTI, alongside extended neurodevelopmental follow-up, may help elucidate the timing and mechanisms underlying these associations and support improved early risk stratification and intervention.

## Supplementary Information

Supplementary Material 1

Supplementary Material 2

## Data Availability

The data that support the findings of this study are not openly available due to reasons of sensitivity and are available from the corresponding author upon reasonable request.
