# Supplementary material for: Opioid exposure on the preterm brain: qualitative and quantitative MRI analysis
Source: Intensive Care Med Paediatr Neonatal. 2025 Dec 29;4(1):4. doi: 10.1007/s44253-025-00105-1 (PMC12858512; doi:10.1007/s44253-025-00105-1)
Supplement: Supplementary file 1 — Supplementary Material 1 [file 44253_2025_105_MOESM1_ESM.pdf]

<u>Supplemental Information 1 – Guideline MRI procedures</u>

Please note that the guideline below was previously published by Plaisier et al.
For more information, refer to the original paper (1)

TAILORED GUIDELINE FOR MRI PROCEDURES IN PRETERM INFANTS
'Safety of routine early MRI in preterm infants' – Pediatric Radiology

Preparation

The medical team (attending neonatologist, pediatric radiologist and nursing staff) decides whether the infant is medically stable enough to undergo an MRI scan and whether the scan is indicated.
A multidisciplinary approach with close communication is essential.
A checklist is used to prepare the infant and equipment for the procedure; this checklist ensures a minimal risk of adverse events related to incorrect execution of the procedure.
An MR-compatible incubator is used, which provides controlled temperature and humidity as well as MR-compatible pulse oximetry and ventilation. The MR-compatible incubator is checked as follows: the temperature is set as it is set on the infant's own incubator; nonmagnetic air and oxygen tanks are present with sufficient capacity; and equipment for ventilation support is available and working.
A resuscitation bag with all necessary equipment for acute interventions is checked and available during the procedure.
All devices attached to the infant and implants (e.g., ECG leads, pulse oximetry probe, temperature probe, intravascular catheters, ductus arteriosus clips and ventriculo-peritoneal shunts as well as metal-containing infant clothing and bracelets) are checked for MR compatibility (http://www.mrisafety.com). MR-compatible ECG electrodes and pulse oximetry probe are attached to the infant to monitor heart rate and oxygen saturation during the scan.
The infant is protected against noise with moldable earplugs and neonatal earmuffs (Minimufs, provide around 7 Db attenuation).
Infusion lines are sufficiently extended such that the infant can undergo an MRI scan while the infusion pumps remain outside the scanning room, or MR-compatible infusion pumps can be used.
The infant is placed in the MR incubator in a comfortable and secure way with small cushions to encourage sleep and reduce movement. As sedation can cause respiratory and circulatory compromise, we do not use sedation in preterm infants.

Transport

A time-out procedure is performed before leaving the NICU such that a quick re-check is conducted and all involved parties agree on the following: the correct infant has been properly prepared, the MR incubator is set correctly, the infant is stable and comfortable and the MR department is ready to scan the infant.
Transport is accompanied by trained staff, and physiological stability is monitored during transport.

During the acquisition

Staff trained in neonatal life support remain present throughout the MRI scan.
A room near the MR suite with equipment, supplies and guidelines for neonatal resuscitation is checked and available during the MRI scan.

The technician at the MR suite performs a metal check on the infant, incubator, oxygen and air tanks and accompanying staff before entering the MR suite. Because of the potential hazards associated with the strong electromagnetic field, MR safety training for all accompanying staff is recommended and provided in our setting.
Before the actual MR procedure starts, the presence of adequate respiratory support, hemodynamic stability and the infant's comfort are verified.
Hemodynamic stability is closely monitored from the MRI incubator's screen, which can be seen from the console room. The MRI procedure should be interrupted if hemodynamic stability is compromised or if there is any doubt about it.

After the MRI scan
After the acquisition, the infant's hemodynamic stability and respiratory support are checked again before returning to the NICU.
Upon arrival to the NICU, a handover of the procedure to medical and nursing staff takes place, and possible adverse events are noted.
The MR-compatible incubator and accessories are cleaned, and the resuscitation bag is refilled if necessary.
During the subsequent 24 hours, the infant's vital parameters and hemodynamic stability are monitored continuously.

Supplemental Information 2 MRI - imaging protocol

The imaging protocol included the following: axial and sagittal T1-weighted spin echo, axial T2-weighted dual spin echo, axial 3-D T1-weighted SPGR and echo planar diffusion tensor imaging. For quantitative analyses, we used axial fast-spin echo (FSE) T2-weighted scans (TR = 13000 ms, TE = 139 ms, matrix size = 256x256, voxel size = 0.7 mm x 0.7 mm x 1.2 mm, flip angle = 90°).

Supplemental Information 3 - Brain volume calculation

Commented [Sd1]: Nog benoemen dat dit in ook in een ander stuk staat? Van Fotis? Zoiets als hieronder?

Please note that the description below will also be published as a supplemental file in a paper by F. Savvopoulos et al. Manuscript in preparation.

The T2-weighted volume of each subject was rigidly registered (6-degrees of freedom) to an age-matched template using FSL's FLIRT (v6.0.3), resulting in volumes with 0.85 mm isotropic voxels. This step was taken to correct motion effects and align all brains to a common spatial orientation. The rigid transformation involved only translations and rotations, thus preserving individual differences between subjects. The motion corrected data was then used as input for the dHCP structural pipeline. Brain volumes were calculated using in-house software written in Python (v3.9) and utilizing functions from the Connectome Workbench (v1.5). First, a custom color-map was applied to the volume segmented label image for visualization and quality control (QC) purposes. Second, each volume within the label image was extracted as an individual region of interest (ROI). Third, individual volumes were calculated by counting the voxels within each ROI and then multiplying this number by the voxel size (in mm^3).

We assessed the anatomical accuracy of the derived volumes by overlaying the segmented volume onto its corresponding T2-weighted volume, which was used as the ground truth. Working slice by slice and adjusting the transparency of the image, we evaluated the extent of anatomical alignment between the segmented volume and the original anatomy. Slight overestimations or underestimations in the segmented volumes were corrected with ITK-SNAP (v.4.0.1). This corrective step was implemented on fifteen out of the sixty-three available volumes. Notably, we observed that cerebellar segmentation posed the most significant challenge and necessitated the highest number of corrections. In instances of pronounced over- or underestimation, the cerebellar volume was excluded after evaluation by three researchers.

1. Plaisier A, Raets MM, van der Starre C, Feijen-Roon M, Govaert P, Lequin MH, et al. Safety of routine early MRI in preterm infants. Pediatr Radiol. 2012;42(10):1205-11.
