# Supplementary material for: Opioid exposure on the preterm brain: qualitative and quantitative MRI analysis
Source: Intensive Care Med Paediatr Neonatal. 2025 Dec 29;4(1):4. doi: 10.1007/s44253-025-00105-1 (PMC12858512; doi:10.1007/s44253-025-00105-1)
Supplement: Supplementary file 2 — Supplementary Material 2 [file 44253_2025_105_MOESM2_ESM.pdf]

Supplemental information 4

Table SI2 Cognitive and motor outcomes in infants who were or were not exposed to opioids

	**Total cohort (n = 280)**						**Opioids pre-TEA (n = 93)**						**No opioids pre-TEA (n = 187)**						
	**n**	**mean**	**min-max**	**SD**	**difference from norm**	**missing (%)**	**n**	**mean**	**min-max**	**SD**	**difference from norm**	**missing (%)**	**n**	**mean**	**min-max**	**SD**	**difference from norm**	**missing (%)**	**difference between groups**
**Bayley-III**																			
Corrected age, *months*		23.8		1.1				23.9		1.2				23.9		1.0			
Cognitive score	215	97.5	65-140	11.6	**p = 0.002**	(23.2)	62	94.2	65-115	11.8	**p < 0.001**	(33)	153	98.8	65-140	11.3	p = 0.21	(18)	**p = 0.008**
Motor score	154	99.5	67-129	10.5	p = 0.517	(45)	48	97.8	67-124	11.3	P = 0.174	(48)	106	100.2	73-129	10.1	p = 0.83	(43)	p = 0.19
WPPSI-III																			
Age, *years*		5.3		0.5				5.2		0.5				5.4		0.5			
Full-scale IQ	133	94.2	60-127	14.4	**p < 0.001**	(53)	42	95.4	60-127	15.0	**p = 0.026**	(55)	91	93.8	61-124	15.0	**p < 0.001**	(51)	p = 0.555
Verbal IQ	133	97.6	57-137	15.0	p = 0.071	(53)	42	99.8	76-137	13.5	p = 0.937	(55)	91	96.6	57-134	15.5	**p = 0.041**	(51)	p = 0.252
Performance IQ	133	96.6	67-126	12.5	**p = 0.002**	(53)	42	97.3	67-118	11.1	p = 0.117	(55)	91	96.3	72-126	13.2	**p = 0.009**	(51)	p = 0.677
Processing speed	130	87.8	55-121	14.3	**p < 0.001**	(54)	41	87.4	62-115	12.9	**p < 0.001**	(56)	89	87.9	55-121	14.9	**p < 0.001**	(52)	p = 0.831
**M-ABC 2**																			
Age, *years*		5.90		0.3				5.9		0.3				5.9		0.3			
Total score	66	7.9	1-15	3.6	**p < 0.001**	(76)	23	6.4	1-13	4.1	**p < 0.001**	(75)	43	8.8	2-15	3.0	p = 0.014	(77)	**p = 0.007**
Manual dexterity	66	7.8	1-16	3.2	**p < 0.001**	(76)	23	6.8	1-13	3.7	**p < 0.001**	(75)	43	8.3	2-16	2.7	p < 0.001	(77)	p = 0.063
Ball skills	66	8.6	1-15	2.9	**p < 0.001**	(76)	23	7.6	1-13	3.1	**p < 0.001**	(75)	43	9.1	4-15	2.7	p = 0.040	(77)	**p = 0.034**
Balance skills	66	8.8	1-16	3.5	**p = 0.005**	(76)	23	7.0	1-14	3.7	**p < 0.001**	(75)	43	9.7	4-16	3.0	p = 0.537	(77)	**p = 0.001**

TEA: term equivalent age, WPPSI: Wechsler's Preschool and Primary Scale of Intelligence, IQ: intelligence quotient. Bold: significant difference. Norm: 100.
